# Microencapsulation Improved Fumaric Acid and Thymol Effects on Broiler Chickens Challenged With a Short-Term Fasting Period

**DOI:** 10.3389/fvets.2021.686143

**Published:** 2021-10-15

**Authors:** Nedra Abdelli, José Francisco Pérez, Ester Vilarrasa, Diego Melo-Duran, Irene Cabeza Luna, Razzagh Karimirad, David Solà-Oriol

**Affiliations:** ^1^Animal Nutrition and Welfare Service (SNIBA), Department of Animal and Science, Facultat de Veterinària, Universitat Autònoma de Barcelona, Barcelona, Spain; ^2^FARMFAES-TECNOVIT, Alforja, Spain; ^3^Department of Animal Science, Lorestan University, Khorramabad, Iran

**Keywords:** microencapsulation, fumaric acid, thymol, gut health, broiler

## Abstract

The first objective of this study was to demonstrate the usefulness of the microencapsulation technique to protect fumaric acid and thymol, avoiding their early absorption and ensuring their slow release throughout the gastrointestinal tract (GIT). For this purpose, the release of a lipid matrix microencapsulated brilliant blue (BB) was assessed *in vitro*, using a simulated broiler intestinal fluid, and *in vivo*. *In vitro* results showed that more than 60% of BB color reached the lower intestine, including 26.6 and 29.7% in the jejunum and ileum, respectively. The second objective was to determine the effects of microencapsulated fumaric acid, thymol, and their mixture on the performance and gut health of broilers challenged with a short-term fasting period (FP). One-day-old male ROSS 308 chickens (*n* = 280) were randomly distributed into seven treatments, with 10 replicates of four birds each. Dietary treatments consisted of a basal diet as negative control (NC), which was then supplemented by either non-microencapsulated fumaric acid (0.9 g/kg), thymol (0.6 g/kg), or a mixture of them. The same additive doses were also administered in a microencapsulated form (1.5 and 3 g/kg for the fumaric acid and thymol, respectively). At day 21, chickens were subjected to a 16.5-h short-term FP to induce an increase in intestinal permeability. Growth performance was assessed weekly. At day 35, ileal tissue and cecal content were collected from one bird per replicate to analyze intestinal histomorphology and microbiota, respectively. No treatment effect was observed on growth performance from day 1 to 21 (*p* > 0.05). Microencapsulated fumaric acid, thymol, or their mixture improved the overall FCR (feed conversion ratio) and increased ileal villi height-to-crypt depth ratio (VH:CD) (*p* < 0.001) on day 35 of the experiment. The microencapsulated mixture of fumaric acid and thymol increased cecal abundance of Bacteroidetes, Bacillaceae, and Rikenellaceae, while decreasing that of Pseudomonadaceae. These results indicate that the microencapsulation technique used in the current study can be useful to protect fumaric acid and thymol, avoiding early absorption, ensure their slow release throughout the GIT, and improve their effects on fasted broiler chickens.

## Introduction

Intensive genetic selection has led to vast improvements in the efficiency of the poultry industry. However, a faster growth rate under intensive conditions coupled with increasing restrictions on the use of antimicrobials is pushing chicken rearing to higher prevalence of intestinal diseases. The gastrointestinal tract (GIT) is a highly complex and dynamic ecosystem involving the qualitative and quantitative equilibrium of the microbial load, morphological structure of the intestinal wall, and the adequate activity of the immune system. An optimal gastrointestinal function is crucial for a sustainable, cost-effective animal production (1). Therefore, the “gut health” concept has drawn significant attention among scientists (2) to develop nutritional strategies and natural alternatives aiming to modulate the gut function toward a satisfactory poultry performance and feed efficiency.

Among the most studied alternatives, organic acids (OAs) and phytogenic feed additives, including essential oils (EOs), may show antimicrobial potential to control dysbiosis and enhance performance of broilers raised without antimicrobials. Although most of the beneficial effects of OAs are associated with their ability to lower the pH, they may also elicit direct non-pH effects on bacterial metabolism by targeting the cell wall, and the cytoplasmic membrane, as well as function related to prokaryote replication and protein synthesis (3). Several studies reported an antimicrobial activity of OAs against most common poultry pathogens such as *Clostridium perfringens* (4), *Salmonella* (5, 6), *Campylobacter jejuni* (7), and *Escherichia coli* (8). On the other hand, there are also published reports that suggest that dietary EOs may stimulate digestive secretions for enhancing nutrient digestibility (9), regulate the gut microbiota composition (10), maintain intestinal integrity, and strengthen mucosal barrier function (11), improve cellular and humoral immunity (12, 13), as well as modulate the immunity related gene expression of chickens (14). The antimicrobial effect of EOs has been linked to their ability to affect the proteomes and cell morphology of pathogenic bacteria (15), which is able to disrupt the outer membrane lipids, and initiate cell lysis leading to an increased permeability. Moreover, combinations of EOs with OAs may show a synergetic potential (16). Nevertheless, the positive effects of OAs and EOs remain controversial (17, 18), which may be attributed to an early absorption of the active compounds that may reduce their levels in the lower GIT (19, 20). Therefore, researchers aim to develop strategies to preserve feed additives from early absorption or volatilization, and to ensure their progressive delivery along the lower GIT. Among the techniques used for protecting feed additives, microencapsulation has been widely applied. In this context, multifarious strategies have been successfully investigated to manufacture microcapsules including chemical methods, such as interfacial polycondensation, interfacial cross-linking, and matrix polymerization; physicochemical methods, such as ionotropic gelation, coacervation-phase separation, chilling, and freeze drying; and physical methods, such as pan coating, air-suspension coating, and centrifugal extrusion (21–23). Despite the benefits of these methods, they still present some drawbacks that may limit their use, such as being costly and time consuming. However, the electrohydrodynamic processes used in the current study is a technique composed of two sister technologies including electrospraying and electrospinning, which provides a broad range of benefits. It is considered as an innovative, cost-effective, and one-step method that ensures the scale-up processes for high-throughput production. Moreover, this energy-saving technique has recently emerged as a promising approach suitable for incorporation of heat-sensitive active compounds (24) by preserving their structure and efficacy upon processing, storage and delivery (21). On the other hand, vegetable oils included in the lipid matrix microparticles used in the current study are composed of long-chain triglycerides reported to possess a slower digestion than that of proteins and polysaccharides (25).

Therefore, we hypothesized that microencapsulation of fumaric acid and thymol, as examples of OAs and EOs, will promote a delayed release of the contained active compounds into the targeted GIT section, exerting beneficial effects on performance, immunity, and the digestive GIT functions in broiler chickens. We also hypothesized that these effects will be more pronounced in broilers exposed to challenging conditions that can negatively affect their gut health.

Therefore, the objectives of this study were (1) to show evidence of the progressive release of fumaric acid and thymol, as examples of OAs and EOs, when these are microencapsulated in lipid matrix microparticles under *in vitro* and *in vivo* intestinal conditions; and (2) to evaluate the effect of microencapsulated fumaric acid and thymol on the performance and gut health of broiler chickens challenged with a short-term fasting period (FP) (as a model of mucosal damage and increased GIT permeability).

## Materials and Methods

### Release of Blue Brilliant (BB) Color

#### *In vitro* Screening

A simulated GIT *in vitro* test was designed to study the release of a microencapsulated BB color (E133) along the GIT of broiler chickens. This microencapsulated BB included 20% of free blue color protected with the same wall material (hydrogenated fats) used to microencapsulate the feed additives tested in the *in vivo* experiment.

The first step was the preparation of a calibration curve. Quantities of 0.0125, 0.025, 0.05, 0.075, 0.1, 0.1125, 0.15, 0.175, and 0.2 g of non-microencapsulated powdered BB color were placed in conical flasks (four for each dose). The following reagents were then added to each flask: 25 ml of phosphate buffer (0.1 M, pH 6.0), 10 ml of 0.2 M HCl, 1 ml of a freshly prepared pepsin, 0.5 ml of chloramphenicol solution, 27 ml of Trizma-Maleate buffer (0.1 M, pH 7.5), 0.5 ml of CaCl_2_ at 325 mM, 1.5 ml of NaCl at 3.25 mM, 0.25 g of bile salts, and 3 ml of pancreatin. Flasks were closed and stirred gently for 2 h at 39°C, and then absorbance was measured using a spectrophotometer at 450 nm. The calibration equation obtained was *Y* = 725.14*X*−0.1887, where “*Y*” was the absorbance at 450 nm and “*X*” was the concentration, and the *R*^2^ was approximately 0.98.

Afterwards, the BB release was simulated under “stomach” and “small intestine” conditions. The BB release under simulated “stomach” conditions was evaluated according to a protocol adapted from Boisen and Fernández (26). A total of 28 conical flasks were used and a total of 0.5 g of microencapsulated BB containing 20% of non-microencapsulated one was placed in each. Afterwards, 25 ml of phosphate buffer (0.l M, pH 6.0) was added to each flask, followed by a gentle magnetic stirring before adding 10 ml of 0.2 M HCl. The pH was then adjusted to pH 2.0 before adding 1 ml of freshly prepared pepsin and 0.5 ml of chloramphenicol. Finally, the flasks were closed and stirred for 90 min in a thermostatically controlled incubator at 39°C. This time was decided based on the study of Ravindran (27) reporting that the digesta spends 90 min in the upper digestive tract. The equivalent transit time per segment was also adapted from the same study being as follows: 30 min in the crop (0–30 min) and 60 min in the proventriculus/gizzard (30–90 min). Thus, during these 90 min, flasks were taken out (four per time) at 30, 60, and 90 min, and absorbance was measured using a spectrophotometer at 450 nm. The concentration was calculated using the calibration equation, which allowed to calculate the percentage of release per segment of the upper digestive tract.

The BB release was then evaluated under simulated small intestine conditions according to Martin et al. (28). After 90 min, the rest of the flasks were removed, and the rest of the previously mentioned reagents used for the preparation of calibration curve were added. Flasks were then stirred gently for 2 min before adding 3 ml of freshly prepared pancreatin. All flasks were then placed in the incubator for a total of 2 h 20 min, reported to be approximately the total transit time in the lower GIT (27). At the end of the first 30 min, the pH was adjusted to 7. Four flasks were removed at 10, 40, 110, and 140 min post-incubation considered as the equivalent transit time for the duodenum, jejunum, ileum, and cecum, respectively (27). The absorbance was measured using a spectrophotometer at 450 nm and the concentration was determined using the calibration equation, which allowed the calculation of the release percentage per segment of the lower digestive tract.

#### *In vivo* Screening

A total of seven 41-day-old Ross 308 male chickens were used to assess the *in vivo* screening of both non-microencapsulated and microencapsulated (containing 20% of E133) BB color. The chickens received the basal diet supplemented with either 0.6% of non-microencapsulated or 3% of microencapsulated BB color (one and six chickens, respectively) during 24 h before being electrically stunned and euthanized. The entire GIT was then removed for the assessment of BB release. Six chickens were used for the microencapsulated BB color to ensure that the kinetics of release were similar in all birds.

#### *In vivo* Experiment

The study was performed at the animal experimental facilities of the Servei de Granges i Camps Experimentals (Universitat Autònoma de Barcelona; Bellaterra, Barcelona, Spain). The experimental procedure received prior approval from the Animal Protocol Review Committee of the same institution (CEEAH1043R2). All animal housing and husbandry conformed to the European Union Guidelines (29).

#### Experimental Design, Dietary Treatments, and Animal Husbandry

A total of 280 1-day-old male ROSS 308 chickens were purchased form a local hatchery, where they received *in ovo* vaccinations for Marek disease, Gumboro disease, and infectious bronchitis. Upon arrival, chicks were weighed and allotted, according to initial body weight, to seven dietary treatments in a completely randomized design. Each dietary treatment was replicated 10 times in battery brooder cages with four chickens per replicate. A non-medicated (non-antibiotic or anticoccidials drug), corn–soybean meal-based diet was used as the basal diet for all treatments (Table 1). The used feed additives (Tecnovit, Alforja, Spain) including fumaric acid, thymol, and their mixture were tested either under microencapsulated or non-microencapsulated form. The microencapsulated fumaric acid included 60%, while thymol contained 20% of active compounds. The equivalent concentration of active compounds was used for the non-microencapsulated forms. Dietary treatments were then produced by supplementing the basal diet with the tested feed additives as follows: (1) basal diet, negative control group (NC); (2) NC+ 1.5 g/kg of microencapsulated fumaric acid; (3) NC+ 3 g/kg of microencapsulated thymol; (4) NC+ microencapsulated blend of fumaric acid (1.5 g/kg) and thymol (3 g/kg); (5) NC+ 0.9 g/kg non-microencapsulated fumaric acid; (6) NC+ 0.6 g/kg free thymol; and (7) NC+ mixture of non-microencapsulated fumaric acid (0.9 g/kg) and thymol (0.6 g/kg).

**Table 1:** Dietary compositions and nutrient levels (% as fed-basis, unless otherwise indicated) of the basal diet.

	**Starter**	**Growing**
**Ingredient composition, g/kg diet**		
Maize	550	582
Soybean meal 48	303	350
L-lysine HCl	1.2	0.20
DL-methionine	2.4	1.3
Soy oil	8.0	19.0
Palm oil	—	17.0
Full fat soybean	100.0	—
Limestone	10.3	6.3
Dicalcium phosphate	15.7	15.5
Salt	2.0	2.0
Premix^*^	4.0	4.0
Sodium bicarbonate	3.4	2.7
**Calculated composition (%)**		
Dry matter	87.8	87.9
ME (kcal/kg)	2975	3101
Crude protein	22	21
Lysine	1.35	1.18
Methionine	0.59	0.47
Ca	0.95	0.78
Total P	0.65	0.63
Available P	0.45	0.44
**Analyzed composition (%)**		
Dry matter	88.5	88.2
GE, kcal/kg	4100	4300
Crude protein	21.9	21.4
Ether extract	0.43	0.54
Crude fiber	2.9	2.7
Ash	5.8	5.6

(*) *Provided per kg of feed: Vitamin A (retinyl acetate) 10,000 IU; vitamin D3 (cholecalciferol) 4,800 IU; vitamin B1 (Thiamine) 3 mg; vitamin B2 (riboflavin) 9 mg; vitamin B3 (Nicotinamide) 51 mg; vitamin B6 (pyridoxine hydrochloride) 4.5 mg; vitamin B9 (folic acid) 1.8; vitamin B12 (cyanocobalamin) 0.04 mg; vitamin E (DL-α-Tocopheryl acetate): 45 mg; vitamin K3 (Menadione) 3 mg; pantothenic acid (calcium D-pantothenate) 16.5 mg, biotin [D-(+)-biotin] 0.15 mg; choline chloride 350 mg; iron (FeSO_4_) 54 mg; iodine [Ca(IO_3_)_2_] 1.2 mg; zinc (ZnO) 66 mg; manganese (MnO) 90 mg; copper (CuSO_4_) 12 mg; selenium (Na_2_SeO_3_) 0.2 mg; 6-Phytase EC 3.1.3.26: 1500 FYT; Butylated hydroxytoluene (BHT) 25 mg; Colloidal silica 45 mg, Sepiolite 1,007 mg*.

Chickens were given a two-phase feeding program consisting of a starter (day 0–14) and grower (day 15–35). All diets were formulated according to CVB poultry guidelines (30). All diets used were sampled, ground, and stored at 5°C until they were analyzed in duplicate.

The brooder temperature was maintained at 32°C during the first 2 days and was then gradually reduced to 25°C on day 14. Birds were provided with a 24-h light during the first 2 days, 23 h light/1 h darkness program from day 3–10, and 18 h light/6 h darkness through the remainder period (days 11–35). All birds were allowed *ad libitum* access to feed in mash form, as well as fresh water. Birds and housing facilities were inspected twice daily to control general health status, feed, and water availability, temperature, mortality, and any unexpected events, during the experimental period.

#### Growth Performance Evaluation

Body weight (BW) and feed intake (FI) from each replicate cage were recorded on days 0, 7, 14, 21, 28, and 35. Average daily gain (ADG), average daily feed intake (ADFI), and feed conversion ratio (FCR) were calculated. Mortality rates were recorded daily.

#### Short-Term Fasting-Induced Challenge

On day 21, after finishing the productive performance control, the smallest bird in each replicate was removed for stocking density reasons. Afterwards, a short-term FP was performed by removing feeders for 16 h and 30 min. This aimed to induce a challenge, as this practice has been reported as a model to increase intestinal permeability and thereby negatively affect the gut integrity (31).

#### Sampling Procedure and Analyses

Diet proximate analyses were performed following Association of Official Agricultural Chemists methodology (32): dry matter (Method 934.01), crude protein (Method 968.06), crude fat (Method 2003.05), and crude fiber (Method 962.09). Gross energy was determined by an adiabatic calorimeter (IKAC-4000, Janke-Kunkel; Staufen, Germany).

On day 35, the bird with the closest BW to the mean of the cage was stunned using an electrical stunner (Reference: 105523, FAF, France) before being euthanized for tissue sampling. The GIT was immediately dissected and content from the cecum was collected for microbiota sequencing. Ileal tissue was collected to perform the histomorphological analysis.

#### Histomorphological Analysis

Ileal samples of about 5 cm were collected at the midpoint between Meckel's diverticulum and the ileo-cecal junction. Tissue sections (5 μm) were fixed in 4% paraformaldehyde and then embedded in paraffin. Afterwards, the preparations were deparaffinized and hydrated before being subjected to PAS (Periodic acid-Schiff) staining with Schiff's reagent for morphometric analyses and goblet cells count. Samples were analyzed using a light microscope. The morphometric variables measured included villus height (VH), crypt depth (CD), villus height-to-relative crypt depth ratio (VH:CD), and number of goblet cells/100 μm VH (GC). Ten villi were measured for each sample, and only complete and vertically oriented villi were evaluated. The mean from 10 villi per sample was used as the mean value for further analysis. All morphometric analysis was done by the same person, who was blinded to the treatments.

#### Microbial Diversity Analysis

Bacterial DNA was extracted from cecal content samples (250 mg) using the commercial MagMAX CORE Nucleic Acid Purification 500RXN Kit (Thermo Fisher, TX, USA) and following the manufacturer's instructions. For 16S rRNA gene sequence-based analysis, the V3–V4 region of the bacteria 16S ribosomal RNA gene were amplified by PCR (95°C for 3 min, followed by 25 cycles at 95°C for 30 s, 55°C for 30 s, and 72°C for 30 s and 72°C for 5 min) using primers F5′-barcode TCGTCGGCAGCGTCAGATGTGTATAAGAGACAGCCTACGGGNGGCW GCA G-3′ and R5′-GTCTCGTGGGCTCGGAGATGTGTATAAGAGACAGGACTACHVGGGTATCTAATCC-3′. A negative control of the DNA extraction and a positive Mock Community control were included to ensure quality control. After 25 cycles of amplifications, 550-bp amplicons were obtained. The Illumina Miseq sequencing 300 × 2 approach was used. Raw sequencing reads were quality clipped, assembled, and compared with available genomic sequences using a Microomics Systems S.L (Barcelona, Spain) software and were validated and subsequently completed with the Kraken Metagenomics (33) and QIIME (34) software. Taxonomic assignment of phylotypes was performed using a Bayesian classifier trained with Silva database version 132 (99% Operational taxonomic units full-length sequences) (35).

### Statistical Analysis

Statistical analyses were carried out on BW, ADG, ADFI, FCR, and histomorphological analysis with ANOVA using the GLM procedure of SAS software (SAS 9.4 Institute Inc., Cary, NC, USA). Normal distribution and homoscedasticity of variances were checked prior to the analysis, using the Shapiro–Wilk test and Levene's test from UNIVARIATE and GLM procedures, respectively. All data related to growth performance and intestinal histomorphology were firstly analyzed according to a according to a completely randomized design, considering treatment groups as the predictor and the number of cages (individual broiler chickens for the histomorphology) as the experimental unit. A further analysis of contrasts excluding the NC aiming to compare the microencapsulated feed additives to the non-microencapsulated ones was also performed for the growth performance and histomorphological data. Means were compared using the Tukey multiple comparisons test and deemed significant at *p* ≤ 0.05.

Biostatistical analysis for microbiota was performed using open-source software RStudio v.3.5.1. Diversity was analyzed at OTU level using a vegan package (36). Richness and α-diversity were calculated using raw counts based on Simpson, Shannon, and Inverse-Simpson estimators. β-diversity was evaluated by multivariate ANOVA. Finally, differential abundance analysis was performed with taxa relative abundances under a zero-inflated log normal mixture model, and *p*-values were corrected by false discovery rate (FDR) using a metagenomeSeq package (37).

## Results

### Release of Blue Brilliant (BB) Color

#### *In vitro* Screening

**Figure 1:**
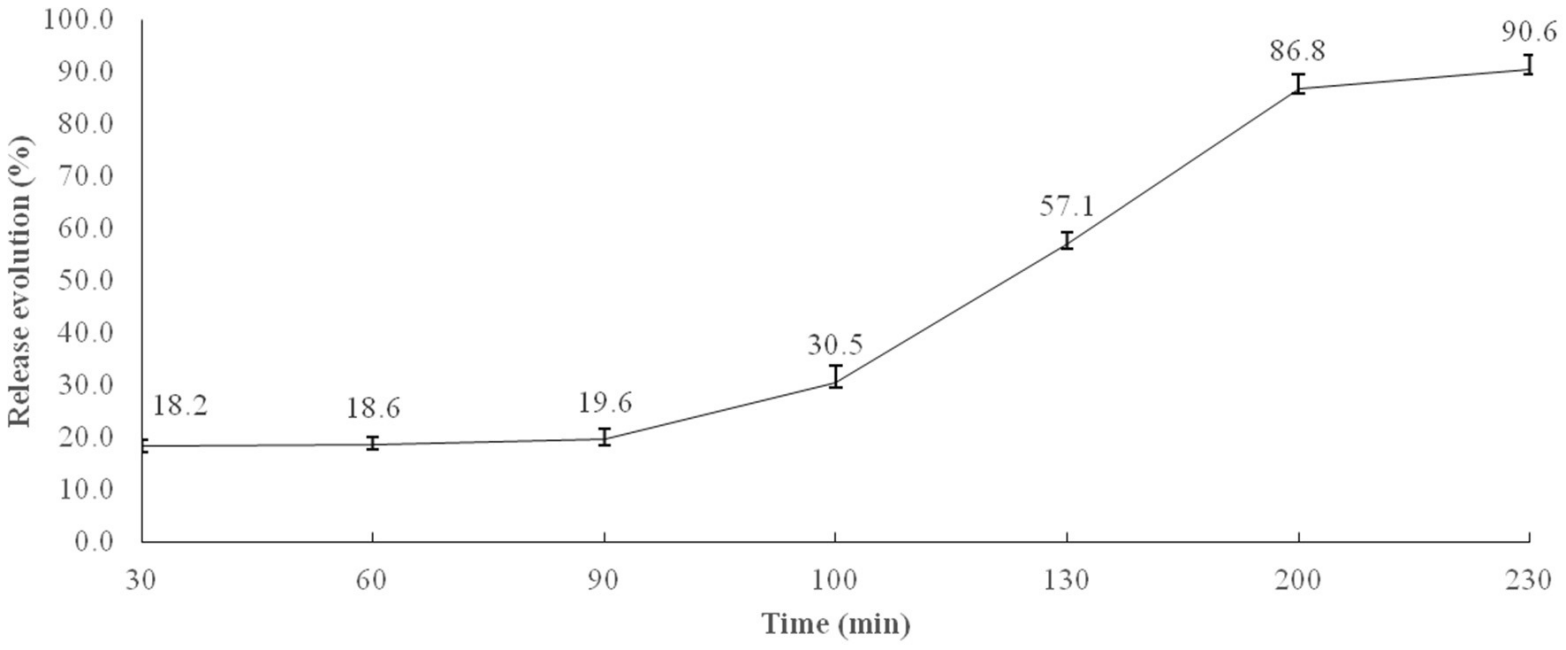
*In vitro* microencapsulated BB release profile in simulated stomach (0–90 min) and small intestine conditions (90–230 min). Each value is the average release of four flasks at simulated conditions of the crop, 0–30 min; gizzard, 30–90 min; duodenum, 90–100; jejunum, 100–130 min; ileum, 130–200 min; and cecum, 200–230 min.

The percentage release of microencapsulated BB was calculated taking into account the transit time (min) in the broiler GIT, adapted from (27). Figure 1 shows the results obtained, indicating that about 19.6% of microencapsulated BB were released in times equivalent to crop and gizzard retention, 10.9% in duodenum, 26.6% in jejunum, 29.7% in ileum, and 3.8% in the cecum.

#### *In vivo* Screening

**Figure 2:**
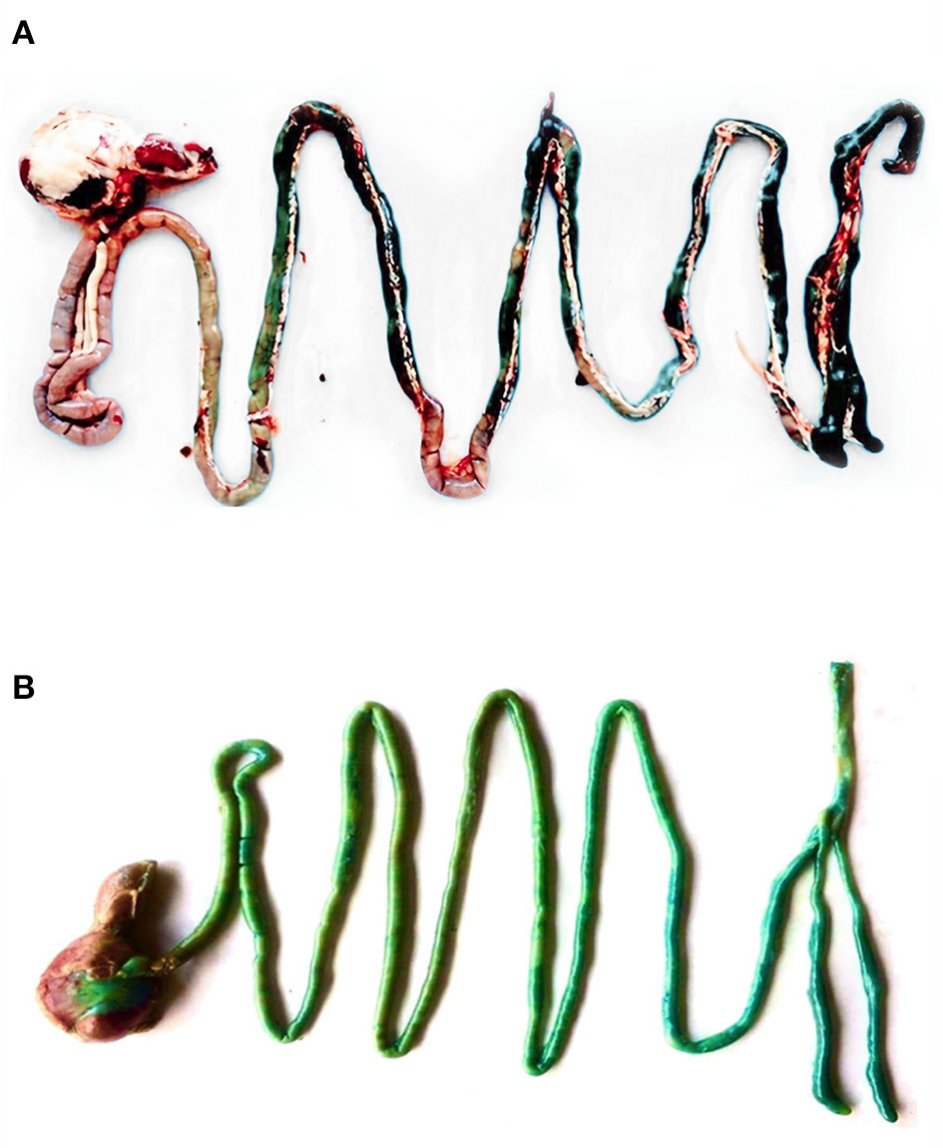
The GIT of a 42-day-old broiler supplemented with either microencapsulated **(A)** or non-microencapsulated BB **(B)**.

Figure 2 shows the GIT of a 42-day-old broiler supplemented by either microencapsulated (A) or non-microencapsulated BB (B). The blue color was observed in the entire GIT of the broiler supplemented with non-microencapsulated BB, whereas it was observed only from the jejunum and backwards for the birds receiving the microencapsulated BB. Unfortunately, the quantification of the *in vivo* release of the microencapsulated BB was not possible. The reason behind this limitation was that once mixed with the feed mostly composed of maize in the GIT, the digesta color turns to green, making the use of the equation of the calibration curve previously established, no longer correct.

### *In vivo* Experiment

#### Growth Performance

Growth performance data are shown in Table 2. No treatment effect was observed on growth performance of broiler chickens before performing the short-term FP challenge on day 21. The FCR from day 21 to day 28 as well as the overall FCR were improved in the experimental groups fed the microencapsulated fumaric acid, thymol, or their mixture (*p* < 0.001). The analysis of contrasts showed an improved overall ADG and FCR by the supplementation of microencapsulated form of all the feed additives (*p* < 0.001) as compared to the non-encapsulated ones and the NC.

**Table 2:** Effect of dietary treatments on growth performance of broiler chickens.

**Items**	**Experimental treatments**							**SEM**	***p***
	**NC**	**Microencapsulated**			**Non-microencapsulated**				
		**OA**	**EO**	**OA+EO**	**OA**	**EO**	**OA+EO**		
**BW g**									
Day 0	42.8	43.2	43.0	42.9	43.3	43.1	42.9	0.16	0.350
Day 21	720	719	699	699	710	705	708	15.6	0.931
Day 28	1106^b^	1266^a^	1240^a^	1260^a^	1129^b^	1112^b^	1132^b^	21.0	<0.001
Day 35	1694^ab^	1802^a^	1790^a^	1804^a^	1670^ab^	1646^b^	1682^ab^	33.2	0.001
**ADG g/day**									
Days 0–21	32.2	32.2	31.2	31.2	31.7	31.5	31.7	0.9	0.501
Days 21–28	55.1^b^	78.1^a^	77.4^a^	80.2^a^	59.7^b^	58.1^b^	60.5^b^	2.24	<0.001
Days 28–35	84.1	76.5	78.6	77.7	77.4	76.4	78.6	4.24	0.892
Days 0–35	47.2^ab^	50.3^a^	49.9^a^	50.3^a^	46.5^ab^	45.8^b^	46.8^ab^	0.95	0.001
**ADFI g/day**									
Days 0–21	44.2	44.1	43	42.3	43.1	42.5	43.2	1.13	0.460
Days 21–28	94.2^b^	111.9^a^	109.2^ab^	115.4^a^	106.3^b^	103.4^b^	102.9^b^	3.64	0.004
days 28–35	143.0	122.4	128.6	124.9	132.6	131.4	133.9	6.97	0.501
Days 0–35	73.1	73.3	73.4	73.5	73.6	72.4	73.3	1.70	0.990
**FCR**									
Days 0–21	1.37	1.37	1.38	1.35	1.36	1.35	1.36	0.040	0.762
Days 21–28	1.71^a^	1.43^b^	1.41^b^	1.44^b^	1.78^a^	1.78^a^	1.70^a^	0.036	<0.001
Days 28–35	1.70^a^	1.60^b^	1.64^ab^	1.61^b^	1.71^a^	1.72^a^	1.70^a^	0.021	0.006
Days 0–35	1.55^a^	1.46^b^	1.47^b^	1.46^b^	1.58^a^	1.58^a^	1.57^a^	0.017	<0.001

*NC, negative control; OA, organic acid: fumaric; EO, essential oil: thymol*.

*Inclusion levels: microencapsulated fumaric acid: 1.5 g/kg; microencapsulated thymol: 3 g/kg; non-microencapsulated fumaric acid: 0.9 g/kg; non-microencapsulated thymol: 0.6 g/kg*.

*Different letters indicate significant difference between the seven dietary treatments at p ≤ 0.05.*

*Data are presented as mean (n = 10 replicates/group for all evaluated parameters)*.

#### Histomorphological Analysis

Histomorphological analysis of the middle portion of the ileum is shown in Table 3. Results showed that the experimental groups receiving either the microencapsulated fumaric acid or the microencapsulated mixture of fumaric acid and thymol exhibited higher VH (*p* = 0.040) than the NC group. All experimental groups fed the microencapsulated feed additives showed lower CD, higher VH:CD ratio, and lower count of goblet cells/100 μm VH (*p* < 0.001).

**Table 3:** Effect of dietary treatments on the ileal histomorphology on day 35 of age.

**Items**	**Experimental treatments**							**SEM**	***p***
	**NC**	**Microencapsulated**			**Non-microencapsulated**				
		**OA**	**EO**	**OA+EO**	**OA**	**EO**	**OA+EO**		
VH	607.8^b^	742.7^a^	720.6^ab^	754.8^a^	639.3^ab^	657.6^ab^	628.1^ab^	28.32	0.040
CD	81.3^ab^	66.6^c^	64.6^c^	64.0^c^	82.9^ab^	76.9^b^	86.1^a^	2.45	<0.001
VH:CD	7.5^b^	11.1^a^	11.2^a^	11.8^a^	7.8^b^	8.6^b^	7.3^b^	0.42	<0.001
Goblet cells/100 μm VH	27.7^a^	14.1^b^	15.4^b^	16.1^b^	24.3^a^	23.5^a^	26.0^a^	0.67	<0.001

*NC, negative control; OA, organic acid: fumaric; EO, essential oil: thymol*.

*Inclusion levels: microencapsulated fumaric acid: 1.5 g/kg; microencapsulated thymol: 3 g/kg; non-microencapsulated fumaric acid: 0.9 g/kg; non-microencapsulated thymol: 0.6 g/kg*.

*Different letters indicate significant difference between the seven dietary treatments at p ≤ 0.05*.

*Data are presented as mean (n = 10 replicates/group for all evaluated parameters)*.

#### Microbial Diversity Analysis

**Figure 3:**
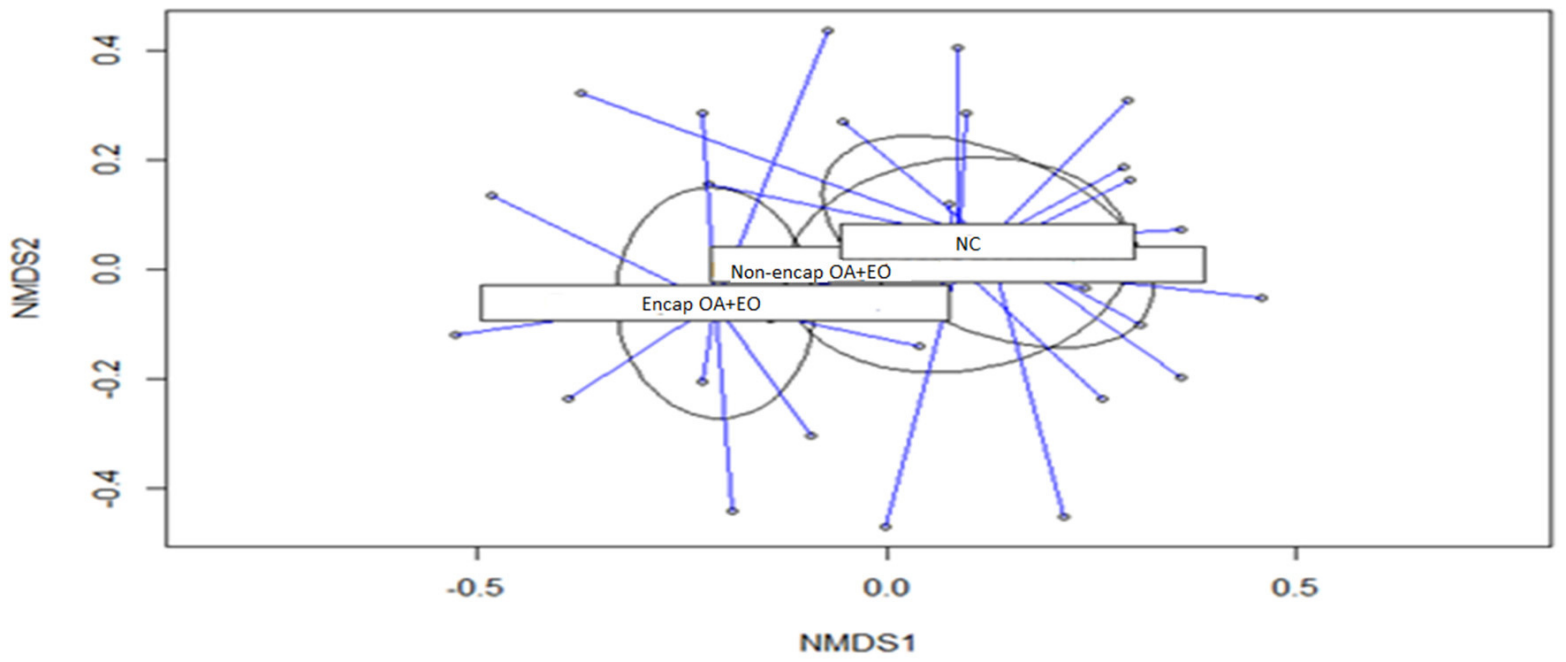
Non-metric multidimensional scaling (NMDS) ordination plot of bacterial communities of the different treatment groups (*p* = 0.430). NC, negative control; OA, organic acid: fumaric; EO, essential oil: thymol. Encap, microencapsulated; Non-encap, non-microencapsulated. Inclusion levels: microencapsulated fumaric acid: 1.5 g/kg; microencapsulated thymol: 3 g/kg; non-microencapsulated fumaric acid: 0.9 g/kg; non-microencapsulated thymol: 0.6 g/kg.

Results of microbial diversity analysis revealed that neither α-diversity (Table 4) nor β-diversity (Figure 3) was different among experimental groups (*p* > 0.05).

**Table 4:** Effect of dietary treatments on microbiota α-diversity indices in cecal content of broiler chickens on day 35.

	**Experimental treatments**			**SEM**	***p***
	**NC**	**Encap OA+EO**	**Non-encap OA+EO**		
Shannon	3.72	3.67	3.60	0.028	0.751
Simpson	0.94	0.94	0.93	0.032	0.962
Inverse Simpson	18.7	16.5	16.4	1.22	0.570

*NC, negative control; OA, organic acid: fumaric; EO, essential oil: thymol*.

*Encap, microencapsulated; Non-encap, non-microencapsulated.*

*Inclusion levels: microencapsulated fumaric acid: 1.5 g/kg; microencapsulated thymol: 3 g/kg; non-microencapsulated fumaric acid: 0.9 g/kg; non-microencapsulated thymol: 0.6 g/kg*.

*p ≤ 0.05 was considered significant*.

**Figure 4:**
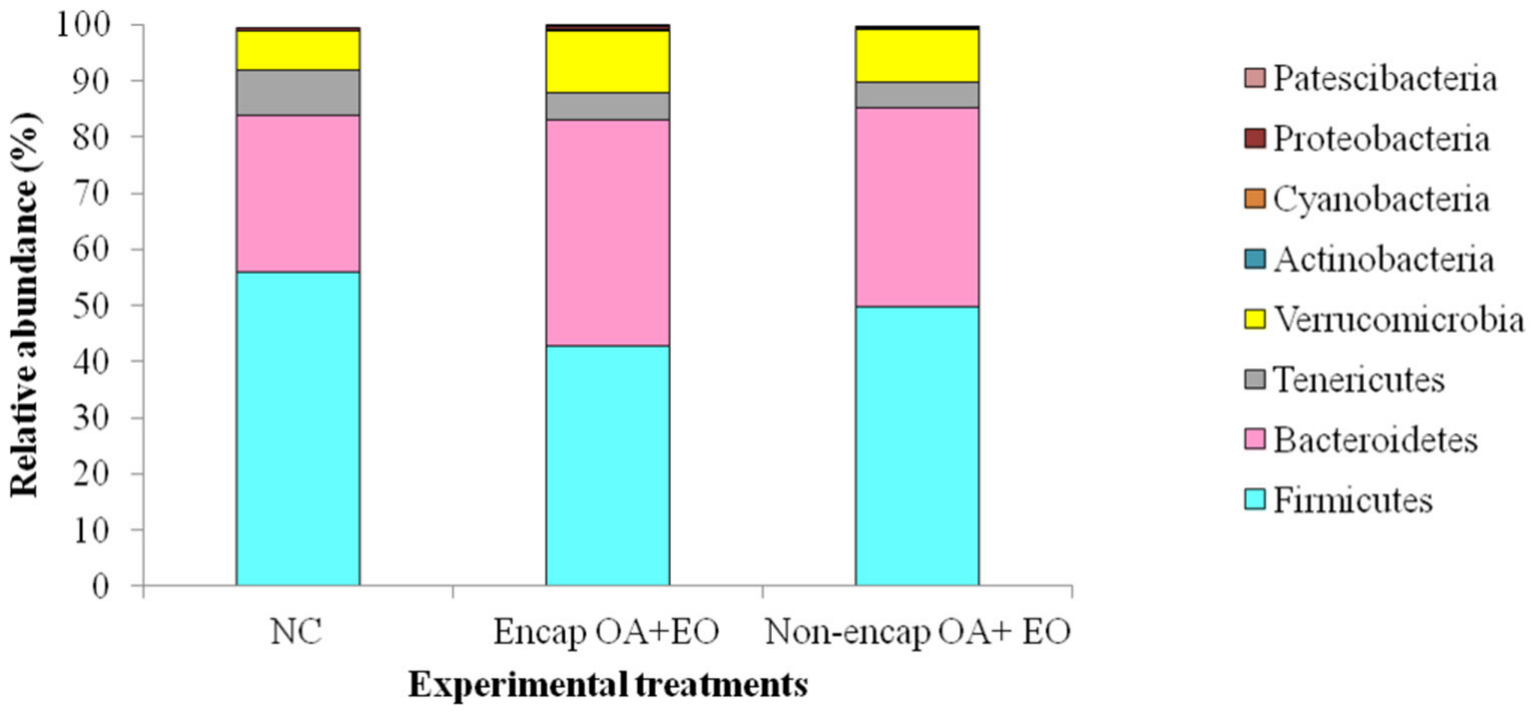
Phyla present in the cecum microbiota of broilers from different treatment groups on day 35. NC, negative control; OA, organic acid: fumaric; EO, essential oil: thymol. Encap: microencapsulated; Non-encap, non-microencapsulated. Inclusion levels: microencapsulated fumaric acid: 1.5 g/kg; microencapsulated thymol: 3 g/kg; non-microencapsulated fumaric acid: 0.9 g/kg; non-microencapsulated thymol: 0.6 g/kg.

**Figure 5:**
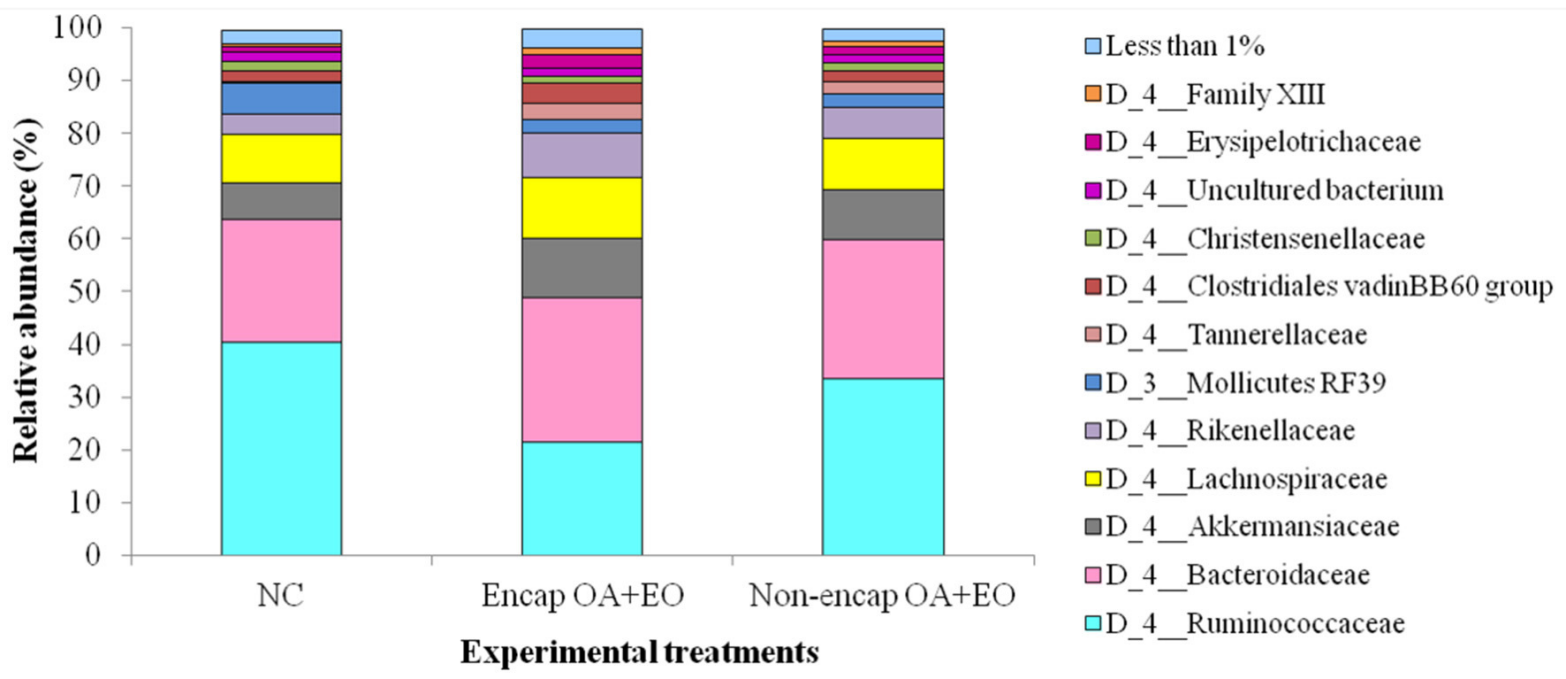
Abundant bacterial families present in the cecum microbiota of broilers from different treatment groups on day 35. NC, negative control; OA, organic acid: fumaric; EO, essential oil: thymol. Encap: microencapsulated; Non-encap, non-microencapsulated. Inclusion levels: microencapsulated fumaric acid: 1.5 g/kg; microencapsulated thymol: 3 g/kg; non-microencapsulated fumaric acid: 0.9 g/kg; non-microencapsulated thymol: 0.6 g/kg.

At the phylum level, eight phyla were determined, including mainly Firmicutes, Bacteroidetes, Tenericutes, and Verrucomicrobia, followed by Proteobacteria, Actinobacteria, Patescibacteria, and Cyanobacteria (Figure 4), with no differences between dietary treatments (*p* > 0.05). The majority of Firmicutes sequences (Figure 5) corresponded to Ruminococcaceae and Lachnospiraceae while the majority of the Bacteroidetes sequences correlated with sequences of Bacteroidaceae and Rikenellaceae.

**Figure 6:**
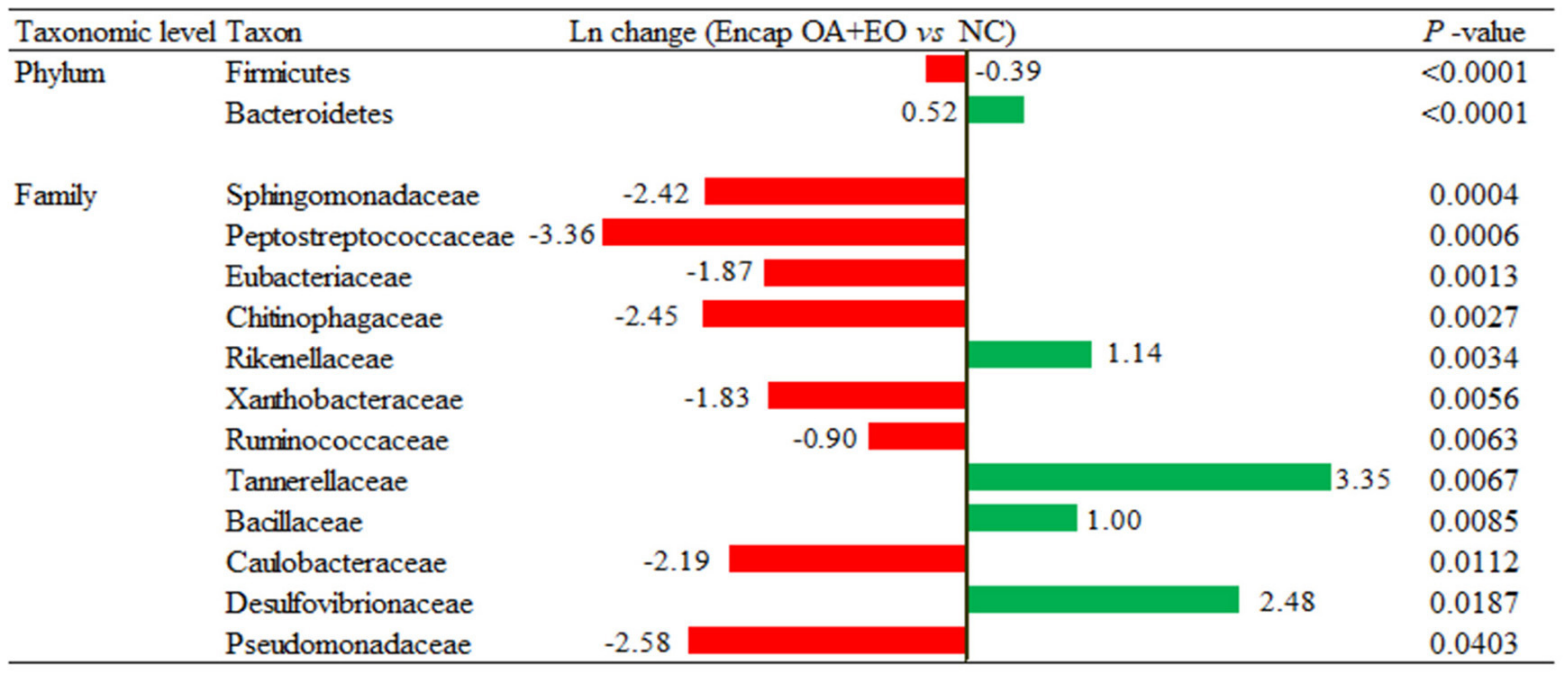
Ln changes promoted by the supplementation of the microencapsulated mixture of fumaric acid and thymol as compared to the NC (fold discovery rate *p*-adjusted <0.05) in taxa. Positive values (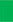) and negative values (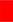) indicate greater and lower abundance. Only significant taxa are presented. Differences presented are based on all taxa detected in samples per diet. NC, negative control; OA, organic acid: fumaric; EO, essential oil: thymol. Encap: microencapsulated. Inclusion levels: microencapsulated fumaric acid: 1.5 g/kg; microencapsulated thymol: 3 g/kg.

A more in-depth examination of the individual metagenomics profile changes was detected on the dietary treatments using log_2_ changes. Broilers supplemented with the microencapsulated mixture of fumaric acid and thymol compared to those fed the NC (Figure 6) had significant differences in the relative abundance of Firmicutes (0.39-fold decrease; *p* < 0.0001) and Bacteroidetes (0.52-fold increase; *p* < 0.0001) phylum, and some families including Rikenellaceae (1.14-fold increase; *p* = 0.0034), Tannerellaceae (3.35-fold increase; *p* = 0.0067), Bacillaceae (1-fold increase; *p* = 0.0085), Chitinophagaceae (2.45-fold decrease, *p* = 0.0027), Pseudomonadaceae (2.58-fold decrease; *p* = 0.0403), and Sphingomonadaceae (2.42-fold decrease; *p* = 0.0004).

**Figure 7:**
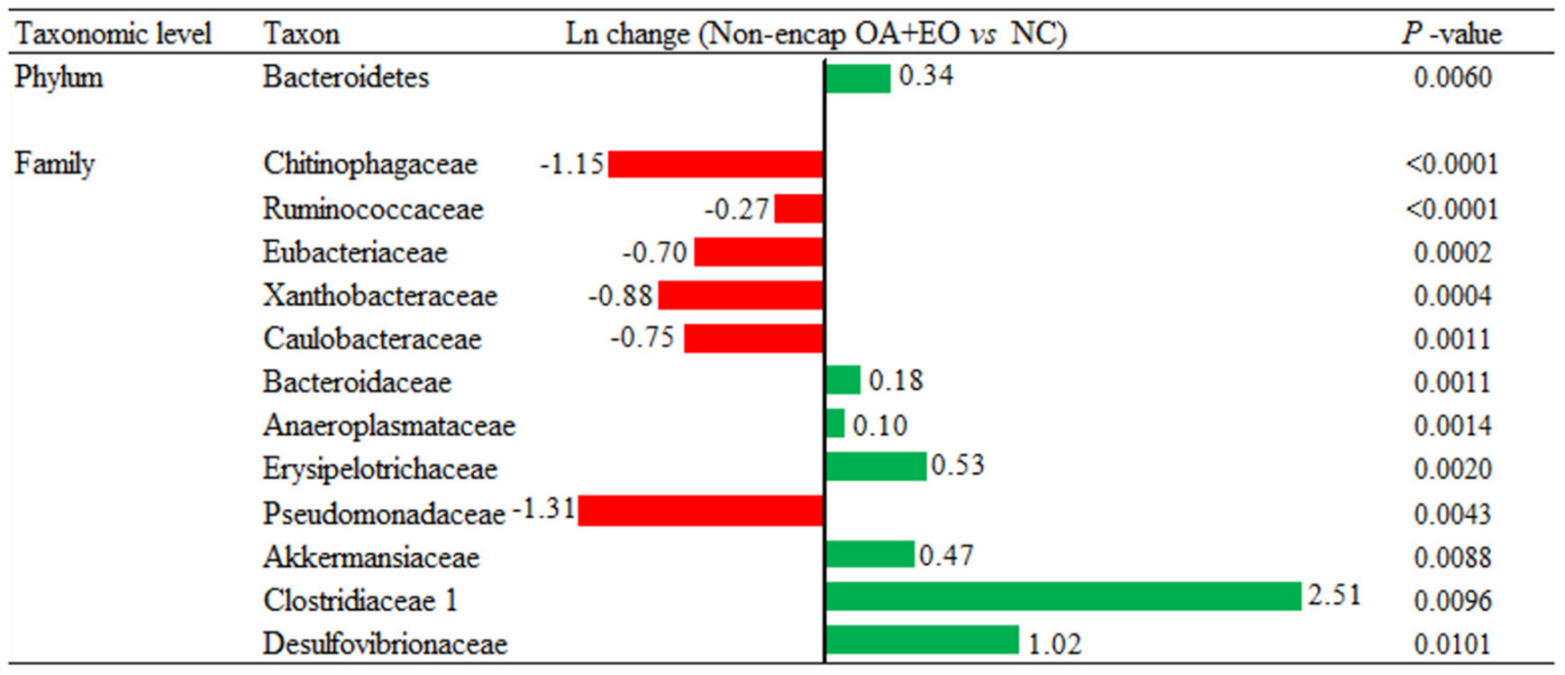
Ln changes promoted by the supplementation of the non-encapsulated mixture of fumaric acid and thymol as compared to the NC (fold discovery rate *P*-adjusted <0.05) in taxa. Positive values (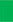) and negative values (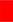) indicate greater and lower abundance. Only significant taxa are presented. Differences presented are based on all taxa detected in samples per diet. NC, negative control; OA, organic acid: fumaric; EO, essential oil: thymol. Non-encap: non-microencapsulated. Inclusion levels: non-microencapsulated fumaric acid: 0.9 g/kg; non-microencapsulated thymol: 0.6 g/kg.

**Figure 8:**
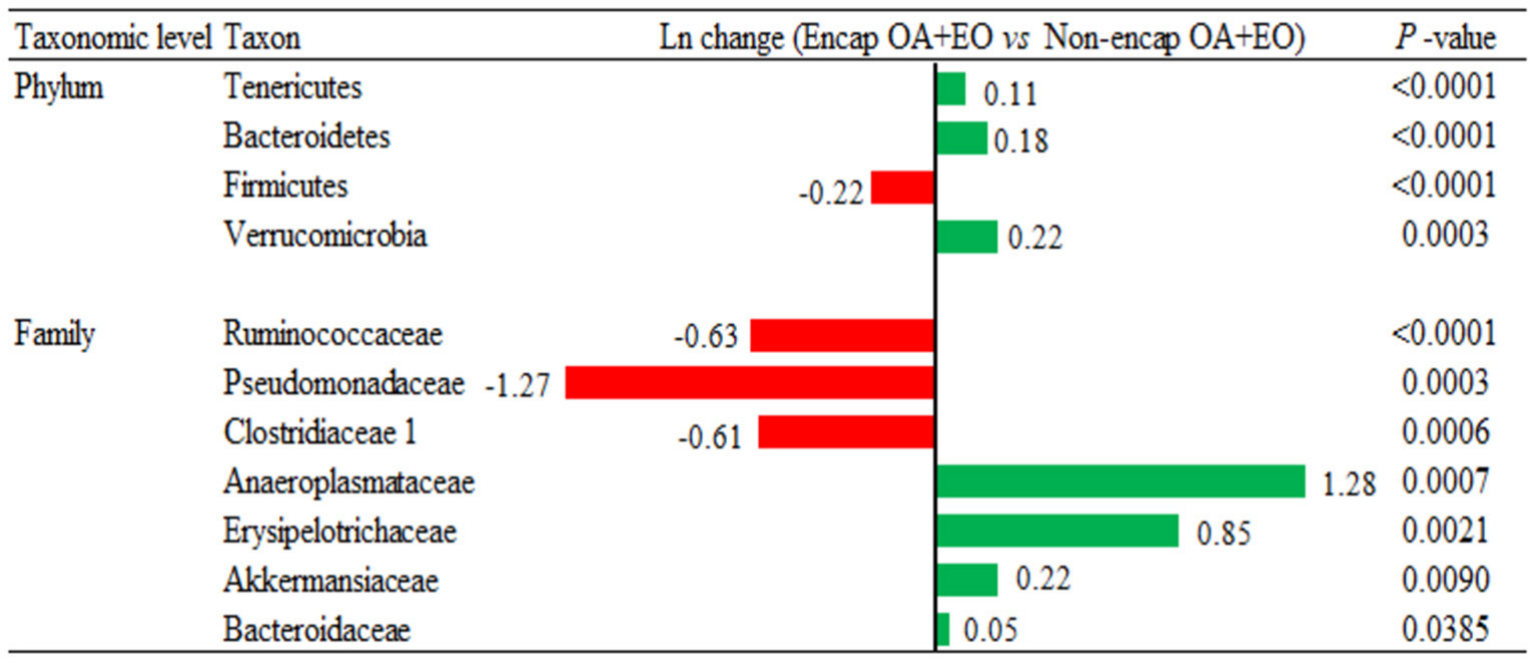
Ln changes promoted by the supplementation of the microencapsulated mixture of fumaric acid and thymol as compared to the non-encapsulated one (fold discovery rate *P*-adjusted <0.05) in taxa. Positive values (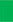) and negative values (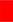) indicate greater and lower abundance. Only significant taxa are presented. Differences presented are based on all taxa detected in samples per diet. NC, negative control; OA, organic acid: fumaric; EO, essential oil: thymol. Encap: microencapsulated; Non-encap, non-microencapsulated. Inclusion levels: microencapsulated fumaric acid: 1.5 g/kg; microencapsulated thymol: 3 g/kg; non-microencapsulated fumaric acid: 0.9 g/kg; non-microencapsulated thymol: 0.6 g/kg.

Broiler supplementation with the non-encapsulated mixture of fumaric acid and thymol significantly changed the abundance of Bacteroidetes (0.34-fold increase; *p* = 0.0060) phyla, and families such as Clostridiaceae 1 (2.51-fold increase; *p* = 0.0096), Erysipelotrichaceae (0.53-fold increase; *p* = 0.0020), Desulfovibrionaceae (1.02-fold increase; *p* = 0.0101), Ruminococcaceae (0.27-fold decrease; *p* < 0.0001), and Chitinophagaceae (1.15-fold decrease; *p* < 0.0001) compared to the NC group (Figure 7). The comparison between both forms of the mixture of fumaric acid and thymol (Figure 8) showed that the microencapsulated one changed the abundance of Firmicutes (0.22-fold decrease; *p* < 0.0001), Bacteroidetes (0.18-fold increase; *p* < 0.0001), Tenericutes (0.11-fold increase; *p* < 0.0001), and Verrucomicrobia (0.22-fold increase; *p* = 0.0003) phylum, and some families like Bacteroidaceae (0.05-fold increase; *p* = 0.0385), Erysipelotrichaceae (0.85-fold increase; *p* = 0.0021), Clostridiaceae 1 (0.61-fold decrease; *p* = 0.0006), and Pseudomonadaceae (1.27-fold decrease; *p* = 0.0003).

## Discussion

In the current study, a short-term FP of 16.5 h was applied on day 21 as an experimental model to challenge gut health. The objective was to investigate whether the evaluated feed additives were able to alleviate the induced negative effects on growth performance, intestinal histomorphology, and microbiota 2 weeks later. Short-term FP up to 24 h has been reported to increase intestinal permeability (38), which may potentially induce bacterial translocation (39), lameness (40), and compromised growth performance (41). A recent study conducted by Herrero-Encinas et al. (31) showed that a 15.5-h short-term FP induced an increase in intestinal permeability by reducing *Claudin-1* expression, which triggered an inflammatory response, resulting in a higher CD and lower VH:CD ratio compared to control non-fasted group.

### Effects of the Free Feed Additives Supplementation

Compared to the NC, the supplementation of non-microencapsulated fumaric acid, thymol, or their mixture did not show any significant effect on growth performance, neither before the short-term FP nor on day 28 or 35. A lack of effect was also obtained with the analysis of ileal histomorphology. However, the significant increase in goblet cells in the ileum of these groups may suggest a higher demand for enhanced mucin secretion, likely helping to reduce the possible damage of the small intestine epithelia (42). Other studies also reported that free OAs failed to reach the cecum in adequate concentrations and, thus, to reduce the *Campylobacter* colonization in broilers (18, 43). This lack of effect may be attributed to their rapid degradation, absorption, and metabolism in the upper section of the GIT (before or almost just entering the duodenum) as shown by the results of the *in vivo* release of non-encapsulated BB. This early absorption means that the majority would not reach the lower GIT tract where they would exert their major functions (18), which may represent a serious limitation for their efficacy.

### Effects of Microencapsulated Feed Additives

The feed additives tested in the current study were microencapsulated using lipid-based particles, reported to possess high encapsulation efficiency, loading capacity, and release efficiency in the small intestine (25). This slow release throughout the GIT was confirmed by the results of the *in vitro* BB release, showing that nearly 60% was released at jejunum, ileum, and cecum equivalent retention times. Although the quantification was not possible, these findings were further supported by the results of the *in vivo* BB release, where the microencapsulated blue color was not observed in the duodenum, suggesting that the release started from the jejunum and backwards. A similar study was performed by Lee et al. (44) to evaluate the physiochemical properties and prolonged release behavior of chitosan-denatured β-lactoglobulin microcapsules for potential food applications. These authors obtained similar promising results, showing that their wall matrix provided both the right timing and location for the BB dye release.

Targeting the lower GIT may be advantageous to enhance the intestinal development, which helps to improve digestion and nutrient absorption, and thereby, the growth performance (19). Growth performances have been evaluated in several experiments in which broiler chickens were supplemented by OAs, EOs, or the mixture of both. However, the considerable increasing number of published articles has generated great information inconsistency. Although some studies revealed improved production performance traits by protected OA supplementation (45), EO supplementation (46), or their mixture (47–49), others reported no effect on growth performance of chickens (50). This discrepancy may be attributed to the heterogeneity of experimental conditions, such as the chemical structure of the OAs or EOs used, the dose, the supplementation form (mixed or not), the sanitary challenge conditions, the number of used chickens, the size of cages or barns, the buffering capacity of feeds, the feed nutritional dietary value, and other factors.

In the current study, the supplementation of microencapsulated fumaric acid, thymol, and their mixture improved the overall FCR during the whole experiment by 5.8% compared to the NC, and up to 7.0% compared to the non-microencapsulated ones. This improvement of growth performance may, in part, be attributed to the observed beneficial effects of these feed additives on ileal histomorphology (increased VH, reduced CD, increased VH:CD ratio, and lower goblet cells/100 μm as compared to the NC group). Similar positive effects were previously reported by other authors, where feeding a protected blend containing a minimum of 200 g/kg of sorbic acid, a minimum of 200 g/kg fumaric acid, a minimum of 100 g/kg thymol to broiler chickens reared under conventional conditions (51) or an encapsulated blend containing 4% thyme, 4% carvacrol, 0.5% hexanoic acid, 3.5% benzoic acid, and 0.5% butyric acid to broiler chickens challenged with necrotic enteritis (48) resulted in longer villi and a greater VH:CD ratio.

In the current study, all the feed additives with the same form of presentation (microencapsulated or non-microencapsulated) showed similar effects on the growth performance and histomorphological analysis. Thus, only the cecal microbiota of the chickens supplemented with the mixture, either microencapsulated or not, has been analyzed as compared to the NC group. The obtained results showed that the supplementation of the microencapsulated mixture of fumaric acid and thymol increased the relative abundance of phyla Bacteroidetes and decreased the relative abundance of phyla Firmicutes compared to the NC group. Similar results were obtained by Chen et al. (10) and Wu et al. (52) by the supplementation of broiler chickens by plant essential oil and sodium butyrate, respectively. However, the supplementation of broilers by a blend containing 4% thyme, 4% carvacrol, 0.5% hexanoic acid, 3.5% benzoic acid, and 0.5% butyric acid encapsulated in Ca-alginate and whey protein microcapsules resulted in an increase of the relative abundance of Firmicutes while the relative abundance of Bacteroidetes decreased (48). Although an increase in fecal Bacteroidetes has been associated with decreased nutrient absorption (53), this phylum composed of Gram-negative bacteria has been recently reported to be gut-friendly, being involved in many important metabolic activities. Indeed, Bacteroidetes participate in the degradation of polysaccharides and other indigestible carbohydrates to produce short-chain fatty acids (SCFAs), especially propionate *via* the succinate pathway (10, 52), utilization of nitrogenous substances, the biotransformation of bile acids, and the prevention of pathogen colonization (54). Among the Bacteroidetes, the microencapsulated blend tested in the current study increased the abundance of Rikenellaceae whose effect on the host gut health remains inconsistent. Some studies considered a reduced abundance of this family to be beneficial (48) as it utilizes the mucin, involved in preventing adhesion of various pathogens and toxins present in the intestinal lumen, as carbon and energy source, which may decrease the intestinal mucosal barrier integrity (55). However, members of this family, such as *Alistipes*, showed to be increased in the current study, have been reported to produce propionic and succinic acids by fermentation of glucose, lactose, mannose, and melibiose, and form the iso-methyl branched-chain fatty acid or long-chain saturated acids (10). Tannerellaceae, a family belonging to Bacteroidetes, which was also increased by the supplementation of the microencapsulated blend of fumaric acid and thymol, produces acetate and succinate as its major metabolic end-products. Succinate can provide energy in two distinct ways. It can be either taken up directly by chicken intestinal cells through a sodium-dependent transport system and then introduced in the tricarboxylic acid or Krebs cycle or converted by several other Bacteroidetes bacteria into propionate after decarboxylation (56). As for Firmicutes, they are Gram-positive bacteria associated with the decomposition of polysaccharides and the production of butyrate (57). Belonging to this phylum, the abundance of Lachnospiraceae, known as butyric acid-forming bacteria (58), showed a numerical increase in the chickens fed the microencapsulated blend of fumaric acid and thymol as compared to the NC group (11.50 vs. 9.18%, respectively). Enhancing SCFA production is crucial for animal gut health. Indeed, butyrate has been reported to possess anti-inflammatory properties through the inhibition of nuclear factor-kappa B activation, leading to decreased expression of pro-inflammatory cytokines (59), which may explain the decrease in necrotic lesions induced by *C. perfringens* in the small intestine (60). It may also improve growth performance through pathogen control (17), barrier integrity enhancement by upregulating the AMP-activated protein kinase, which regulates the assembly of tight junctions (61), and the activation of goblet cells to produce mucin, which forms a protective layer on the enterocytes (62). As for propionate, it can also be used as an energy source by the epithelial cells and is known to stimulate the trypsin activity (63) and to possess health-promoting effects, including an anti-inflammatory activity, which may improve growth performance (56).

On the other hand, the increased abundance of Bacillaceae induced by the microencapsulated blend tested in the current study may be considered beneficial as a recent study showed a positive correlation between this family and total volatile fatty acids (VFAs). Bacillaceae has also been shown to play a key role in improving the immune status by enhancing different antioxidants and tight-junction genes (64).

The increase in the above-mentioned families may explain the decline observed in other families containing pathogen bacteria such as Pseudomonadaceae. The infection of broiler chickens with *Pseudomonas aeruginosa* is associated with high mortality and clinical signs including respiratory manifestations, diarrhea, and septicemia (65). Moreover, *Pseudomonas veronii* is a potential opportunistic pathogen whose abundance increased in broiler chickens challenged by *C. perfringens* (66).

Surprisingly, the microencapsulated blend tested in the current study increased the abundance of Desulfovibrionaceae, a producer of hydrogen sulfides reported to be toxic to mucosal tissue, which leads to severe inflammation of chicken GIT (67) and decreased that of Ruminococcaceae and Peptostreptococcaceae, known as butyric acid-forming bacteria (58). The decrease of Peptostreptococcaceae was not in concordance with previous studies that reported this family to be higher in broiler chickens supplemented with a blend of medium-chain fatty acids containing 0.3% capric acid and 2.7% lauric acid (68), as well as mice supplemented with 13.3 mg/ml of eugenol in drinking water for 7 days (11). However, the improved growth performance and intestinal histomorphology of the supplemented chickens indicated these birds to possess healthier intestinal microbiota compared to the NC group despite the above-mentioned unexpected changes of the gut microbiota.

Taken together, our results indicate that microencapsulating the fumaric acid and thymol using a lipid matrix prevents their absorption in the upper part of the digestive tract and directs their bioactivity toward the lower GIT, mainly the jejunum and ileum. In previous *in vitro* studies, we confirmed that the lipid base particles (empty particles *per se*) did not possess any antimicrobial activity. Therefore, it can be concluded that once released, fumaric acid and thymol enhanced intestinal microbiota balance in favor of beneficial bacteria, which may be responsible for the improvement of ileum histomorphology and thereby feed efficiency of broiler chickens. The positive effects of microencapsulated fumaric acid, thymol, or their combination were observed when broilers were under the challenging conditions of short-term fasting period, but not earlier, highlighting the usefulness of using such feed additives when sanitary conditions of animals are compromised.

## Conclusion

In summary, the results of the current study confirmed the ability of the lipid matrix, obtained through the use of the electrohydrodynamic processes, to allow a slow release of fumaric acid and thymol throughout the broiler GIT. Microencapsulated fumaric acid, thymol, or their combination showed positive effects when broilers were subjected to challenging conditions, alleviating the negative effects promoted by the fasting challenge on animal performance, intestinal histomorpholgy, and microbiota.

## Data Availability Statement

The datasets generated for this study have been submitted to the Sequence Read Archive (SRA) database of the National Center for Biotechnology Information as FASTQ files under study accession number PRJNA734795.

## Ethics Statement

The animal study was reviewed and approved by CEEAH Universitat Autònoma de Barcelona.

## Author Contributions

NA, JFP, EV, and DS-O conceived and designed the study. NA and RK performed the experiments. NA and DM-D analyzed the data. NA wrote the manuscript. JFP, DS-O, EV, and IC corrected the manuscript. All authors read and approved the final manuscript.

## Funding

This research was funded by Tecnología & Vitaminas belonging to the Spanish pharmaceutical company FAES FARMA. NA was funded by an FI Ph.D. grant from the Agència de Gestió d'Ajuts Universitaris i de Recerca de la Generalitat de Catalunya (2018FI_B_01070). DM-D: was funded by a pre-doctoral scholarship from the Secretaria de Educación Superior, Ciencia, Tecnología e Innovación de Ecuador (SENESCYT).

## Conflict of Interest

The authors declare that the research was conducted in the absence of any commercial or financial relationships that could be construed as a potential conflict of interest.

## Publisher's Note

All claims expressed in this article are solely those of the authors and do not necessarily represent those of their affiliated organizations, or those of the publisher, the editors and the reviewers. Any product that may be evaluated in this article, or claim that may be made by its manufacturer, is not guaranteed or endorsed by the publisher.
